# Cardiac Troponin Thresholds and Kinetics to Differentiate Myocardial Injury and Myocardial Infarction

**DOI:** 10.1161/CIRCULATIONAHA.121.054302

**Published:** 2021-06-25

**Authors:** Ryan Wereski, Dorien M. Kimenai, Caelan Taggart, Dimitrios Doudesis, Kuan Ken Lee, Matthew T.H. Lowry, Anda Bularga, David J. Lowe, Takeshi Fujisawa, Fred S. Apple, Paul O. Collinson, Atul Anand, Andrew R. Chapman, Nicholas L. Mills

**Affiliations:** 1 British Heart Foundation Centre for Cardiovascular Science (R.W., C.T., D.D., K.K.L., M.T.H.L., A.B., T.F., A.A., A.R.C., N.L.M.), University of Edinburgh, UK.; 2 Usher Institute (D.M.K., D.D., N.L.M.), University of Edinburgh, UK.; 3 University of Glasgow, School of Medicine, UK (D.J.L.).; 4 Department of Laboratory Medicine and Pathology, Hennepin Healthcare/Hennepin County Medical Center and University of Minnesota, Minneapolis (F.S.A.).; 5 Department of Clinical Blood Sciences and Cardiology, St. George’s University of London, UK (P.O.C.).

**Keywords:** kinetics, myocardial infarction, predictive value of tests, troponin

## Abstract

**Background:**

Although the 99th percentile is the recommended diagnostic threshold for myocardial infarction, some guidelines also advocate the use of higher troponin thresholds to rule in myocardial infarction at presentation. It is unclear whether the magnitude or change in troponin concentration can differentiate causes of myocardial injury and infarction in practice.

**Methods:**

In a secondary analysis of a multicenter randomized controlled trial, we identified 46 092 consecutive patients presenting with suspected acute coronary syndrome without ST-segment–elevation myocardial infarction. High-sensitivity cardiac troponin I concentrations at presentation and on serial testing were compared between patients with myocardial injury and infarction. The positive predictive value and specificity were determined at the sex-specific 99th percentile upper reference limit and rule-in thresholds of 64 ng/L and 5-fold of the upper reference limit for a diagnosis of type 1 myocardial infarction.

**Results:**

Troponin was above the 99th percentile in 8188 patients (18%). The diagnosis was type 1 or type 2 myocardial infarction in 50% and 14% and acute or chronic myocardial injury in 20% and 16%, respectively. Troponin concentrations were similar at presentation in type 1 (median [25th–75th percentile] 91 [30–493] ng/L) and type 2 (50 [22–147] ng/L) myocardial infarction and in acute (50 [26–134] ng/L) and chronic (51 [31–130] ng/L) myocardial injury. The 99th percentile and rule-in thresholds of 64 ng/L and 5-fold upper reference limit gave a positive predictive value of 57% (95% CI, 56%–58%), 59% (58%–61%), and 62% (60%–64%) and a specificity of 96% (96%–96%), 96% (96%–96%), and 98% (97%–98%), respectively. The absolute, relative, and rate of change in troponin concentration were highest in patients with type 1 myocardial infarction (*P*<0.001 for all). Discrimination improved when troponin concentration and change in troponin were combined compared with troponin concentration at presentation alone (area under the curve, 0.661 [0.642–0.680] versus 0.613 [0.594–0.633]).

**Conclusions:**

Although we observed important differences in the kinetics, cardiac troponin concentrations at presentation are insufficient to distinguish type 1 myocardial infarction from other causes of myocardial injury or infarction in practice and should not guide management decisions in isolation.

**Registration:**

URL: https://www.clinicaltrials.gov; Unique identifier: NCT01852123.

## Abstract 2

Supplemental Digital Content is available in the text.

**Clinical Perspective**

**What Is New?**

- In 46 092 consecutive patients with suspected acute coronary syndrome, we evaluated the performance of the 99th percentile rule-in threshold and thresholds of 64 ng/L and 5 times the upper reference limit for the diagnosis of type 1 myocardial infarction.
- Troponin concentrations at presentation have a low positive predictive value for type 1 myocardial infarction, and a threshold 50 times the upper reference limit is required to achieve a positive predictive value ≥70%.
- Change in troponin on serial testing only marginally improves positive predictive value for type 1 myocardial infarction over presenting troponin alone (area under curve, 0.661 [0.642–0.680] versus 0.613 [0.594–0.633]).

**What Are the Clinical Implications?**

- Troponin concentrations at presentation are insufficient to distinguish type 1 myocardial infarction from other causes of myocardial injury or infarction and should not be used in isolation to guide management decisions in patients with suspected acute coronary syndrome.

To promote the adoption of common standards, the Universal Definition of Myocardial Infarction recommends cardiac troponin testing and the 99th percentile upper reference limit (URL) as the diagnostic threshold for myocardial infarction.^1^ In practice, there are many causes of troponin elevation, with about half of all increases attributable to conditions other than type 1 myocardial infarction.^2–9^ Nonetheless, the early differentiation between types of myocardial infarction and acute or chronic nonischemic myocardial injury is important because the immediate management of these conditions differs.^1,10,11^ Prompt treatment with antiplatelet agents, anticoagulation, and coronary revascularization is recommended in type 1 myocardial infarction, whereas these may not be indicated in type 2 myocardial infarction or myocardial injury and may be contraindicated.^8,12^

Alternative thresholds above the 99th percentile have been proposed to improve the positive predictive value (PPV) and specificity of troponin for type 1 myocardial infarction.^11,13–16^ The European Society of Cardiology guidelines propose the use of rule-in thresholds above the 99th percentile to guide admission to cardiology and coronary angiography.^11^ These rule-in thresholds and those 5 times the URL are purported to give a PPV of at least 70% and 90%, respectively.^11^ They were derived in selected patients with chest pain, but, in practice, troponin testing is applied more widely to evaluate patients with suspected acute coronary syndrome presenting with a broader range of symptoms.^3,17^ Guidelines also recommend serial testing with a rise or fall in cardiac troponin needed to confirm the diagnosis of myocardial infarction.^1,10,11,18^ However, patients with type 2 myocardial infarction and acute nonischemic myocardial injury also have dynamic changes in troponin concentration on serial testing.^19–21^ It is unclear whether rule-in thresholds or troponin kinetics can reliably differentiate between types of myocardial infarction or between myocardial injury and infarction in clinical practice.^22^

We aimed to evaluate the performance of recommended cardiac troponin thresholds to rule in the diagnosis of type 1 myocardial infarction at presentation. We also aimed to determine whether the kinetics of cardiac troponin differs sufficiently to discriminate between myocardial injury and infarction.^23^

## Methods

### Transparency and Openness Promotion

The High-STEACS trial (High-Sensitivity Troponin in the Evaluation of Patients With Suspected Acute Coronary Syndrome) makes use of multiple routine electronic health care data sources that are linked, deidentified, and held in a national safe haven that is accessible by approved individuals who have undertaken the necessary governance training. Summary data and the analysis code can be made available on request from the corresponding author.

### Study Population and Trial Design

High-STEACS is a stepped-wedge cluster randomized controlled trial that evaluated the implementation of a high-sensitivity cardiac troponin I assay in consecutive patients with suspected acute coronary syndrome across 10 secondary and tertiary care hospitals in Scotland. A detailed description of this trial has been reported elsewhere,^2^ but in summary, all patients attending the emergency department were screened for suspected acute coronary syndrome by the attending clinician at the time cardiac troponin was requested with the use of an electronic form integrated into the clinical care pathway. Patients were eligible for inclusion if they presented with suspected acute coronary syndrome and had paired cardiac troponin measurements from the standard care and trial assay. Patients were excluded if they had been admitted previously during the trial period or were not residents of Scotland. In this analysis, we excluded patients with ST-segment–elevation myocardial infarction,^24^ those for whom troponin concentration at presentation was missing, or patients for whom the adjudicators were unable to arrive at a consensus for the final diagnosis.

### Measurement of Cardiac Troponin

Cardiac troponin testing was performed at presentation to hospital and was repeated 6 or 12 hours after the onset of symptoms at the discretion of the attending physician in accordance with national guidelines.^25,26^ All patients had troponin measured with a high-sensitivity cardiac troponin I assay (ARCHITECT_*STAT*_ high-sensitive troponin I assay; Abbott Laboratories, Abbott Park, IL). This assay has an interassay coefficient of variation of <10% at 4.7 ng/L and a limit of detection of 1.2 and 1.9 ng/L. For consistency with prior studies, we defined the limit of detection as any concentration <2 ng/L, and for the purpose of this analysis, we assigned concentrations below the limit of detection a value of 1.0 ng/L.^27,28^ The assay has a 99th percentile URL of 26 ng/L, with sex-specific thresholds of 34 and 16 ng/L in men and women, respectively.^29,30^

### Diagnostic Adjudication

All patients with any high-sensitivity cardiac troponin I concentration above the sex-specific 99th percentile were adjudicated and classified according to the Fourth Universal Definition of Myocardial Infarction.^1^ Two physicians independently reviewed all clinical information, with discordant diagnoses resolved by a third physician. Type 1 myocardial infarction was defined as myocardial necrosis (any high-sensitivity cardiac troponin I concentration above the sex-specific 99th percentile with a rise or fall in troponin when serial testing was performed) in the context of a presentation with suspected acute coronary syndrome and symptoms or signs of myocardial ischemia on the ECG. Patients with myocardial necrosis, symptoms or signs of myocardial ischemia, and evidence of myocardial oxygen supply-demand imbalance secondary to an alternative condition without evidence of acute atherothrombosis were classified as having type 2 myocardial infarction.^21^ Patients with elevated troponin concentrations without symptoms or signs of myocardial ischemia were classified as having nonischemic myocardial injury. All nonischemic myocardial injury was classified as acute unless troponin concentrations changed ≤20% on serial testing in accordance with the universal definition or if the adjudicated diagnosis was chronic heart failure or chronic renal failure, for which the classification was chronic myocardial injury. A detailed summary of the adjudication procedures is provided in the Data Supplement.

### Ethics Approval

The study was approved by the Scotland A Research Ethics Committee, by the Public Benefit and Privacy Panel for Health and Social Care, and by each National Health Service Health Board. Individual patient consent was not required, and data from consecutive patients were collected prospectively from the electronic record, deidentified, and linked within secure National Health Service Safe Havens.

### Patient and Public Involvement

Patients and lay representatives were members of the steering committee for the trial and all related studies and were involved in the design, conduct, and approval of the High-STEACS trial.

### Statistical Analysis

Baseline characteristics were summarized for the study population and in groups according to the diagnostic classification: type 1 myocardial infarction, type 2 myocardial infarction, acute myocardial injury, chronic myocardial injury, and no myocardial injury. Group-wise comparisons were performed with χ^2^, Kruskal-Wallis, or 1-way ANOVA tests as appropriate. We constructed confusion matrices and calculated the PPV and specificity for type 1 myocardial infarction of a high-sensitivity cardiac troponin I concentration at presentation above the uniform 99th percentile (26 ng/L), sex-specific 99th percentile (16 ng/L in women, 34 ng/L in men), guideline-recommended rule-in threshold of 64 ng/L, and 3-fold and 5-fold URL thresholds (78 and 130 ng/L, respectively). From prior literature, we also determined the cardiac troponin concentration at presentation that met a prespecified PPV of 75%.^31^ We calculated the 95% CI using a bayesian approach by sampling from a binomial likelihood with noninformative Jeffreys prior (both β-distribution shape parameters equal to 0.5). In a sensitivity analysis, we evaluated the PPV and specificity in patients for whom the primary presenting symptom recorded by the attending clinician was chest pain.

In patients with serial sampling within 12 hours of presentation, we used linear mixed-effects modeling with random slopes and intercepts to evaluate the relationship among symptom onset, troponin, and change in troponin concentration. Nonlinear associations were evaluated by adding a second-order polynomial term for time to the model. We have compared the models with and without a quadratic term for time, and the final model was chosen according to the lowest Akaike information criteria. To illustrate the kinetics of cardiac troponin across the groups, we developed additional models for each diagnostic classification. In each of these models, we included type 2 myocardial infarction or acute or chronic myocardial injury as a fixed effect, with type 1 myocardial infarction as the reference group. To evaluate whether relative or absolute change in troponin on serial testing improves discrimination for type 1 myocardial infarction over troponin concentration at presentation alone, we used logistic regression and compared the area under the receiver-operating characteristic curve. We evaluated models that incorporated relative and absolute changes as continuous measures and absolute and relative delta values of 15 ng/L and 20% as recommended in international guidelines.^10,11^ All analyses were performed in R (version 3.5.1).

## Results

The analysis population comprised 46 092 of the 48 242 patients enrolled in the trial after exclusion of those with ST-segment–elevation myocardial infarction (n=925), those for whom the diagnosis could not be adjudicated (n=1241), and those with missing troponin concentrations at presentation (n=24; Figure I in the Data Supplement).

Cardiac troponin concentrations were above the sex-specific 99th percentile URL in 8188 (18%) patients. The adjudicated diagnosis was type 1 myocardial infarction in 50% (n=4064), type 2 myocardial infarction in 14% (n=1116), acute myocardial injury in 20% (n=1676), and chronic myocardial injury in 16% (n=1287) of patients (Table 1). Patients with type 1 myocardial infarction were younger and more likely to be men than those with type 2 myocardial infarction or acute and chronic myocardial injury. Chest pain was the primary presenting symptom in 90% of patients with type 1 myocardial infarction (3315 of 3692) and 73% of those with type 2 myocardial infarction (744 of 1026) but was less common in patients with acute (38%, 569 of 1495) or chronic (49%, 559 of 1131) myocardial injury.

**Table 1.**
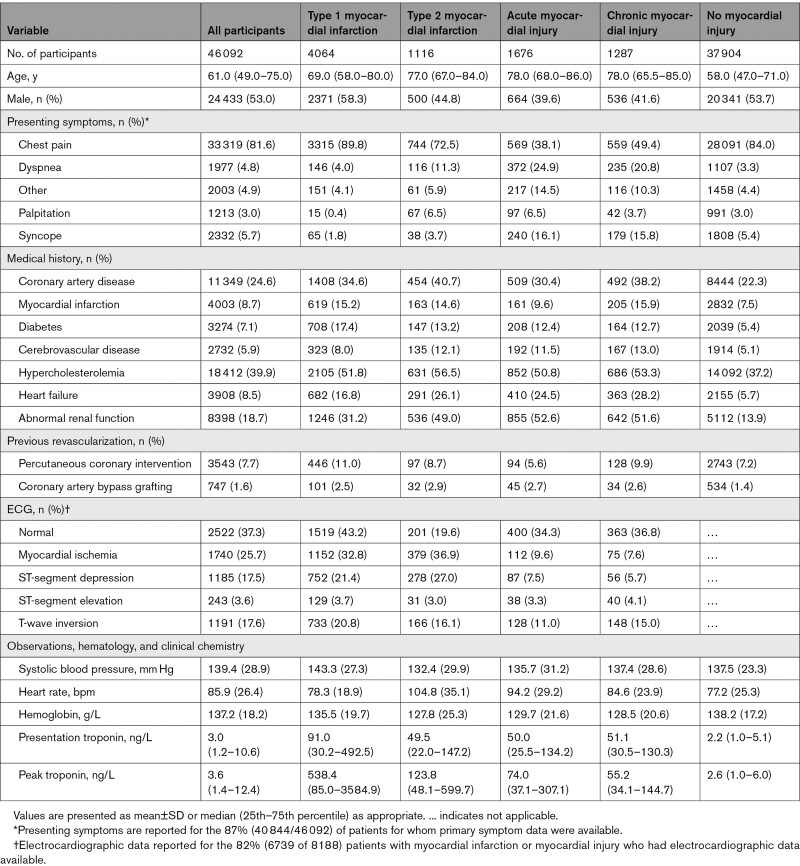
Baseline Characteristics of the Study Population Stratified by Adjudicated Diagnosis of Myocardial Injury or Infarction

### Troponin Concentrations at Presentation in Myocardial Injury and Infarction

**Figure 1.**
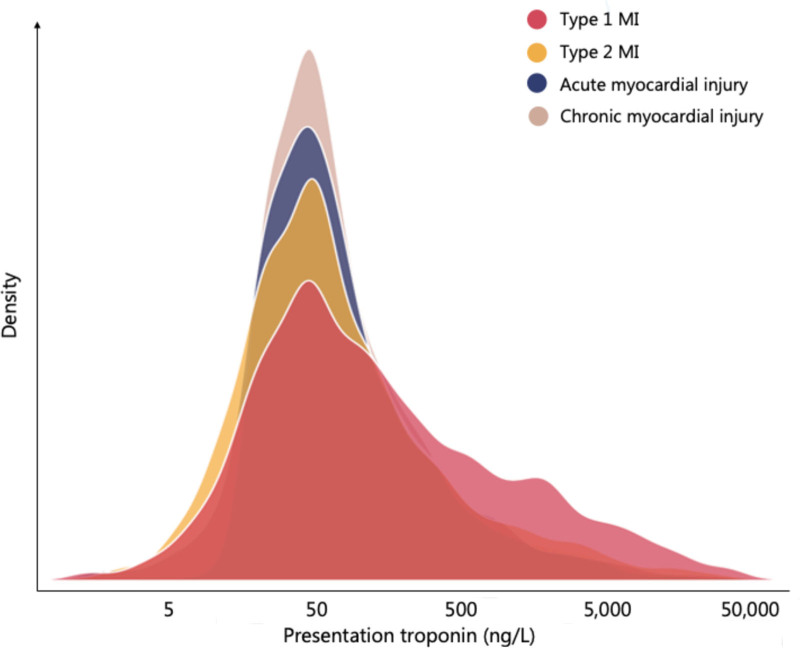
**High-sensitivity cardiac troponin I concentrations at presentation in patients with myocardial injury and infarction.** Kernel density plot of presentation troponin concentration stratified by the adjudicated diagnosis: type 1 myocardial infarction (MI; red), type 2 MI (yellow), acute myocardial injury (blue), and chronic myocardial injury (gray).

**Figure 2.**
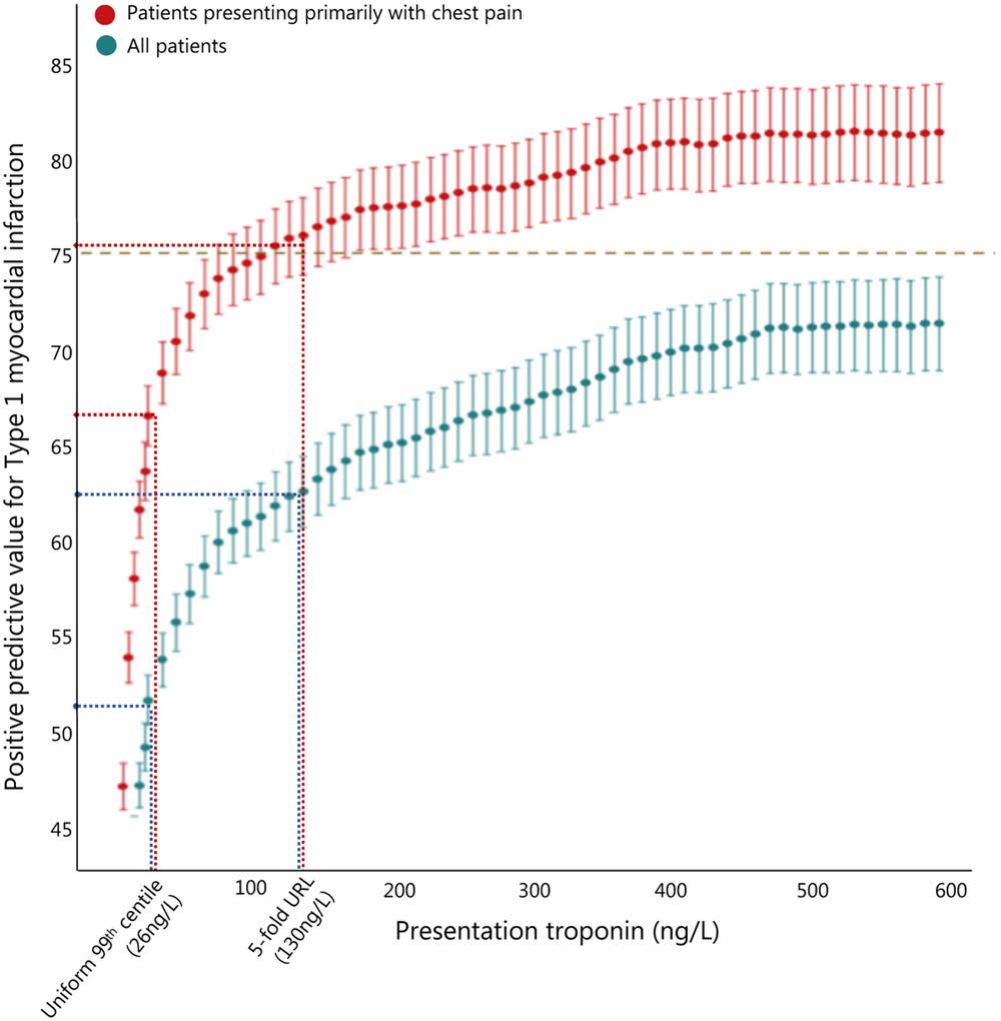
**Positive predictive value of high-sensitivity cardiac troponin I concentration at presentation for a diagnosis of type 1 myocardial infarction.** Positive predictive value and 95% CIs of high-sensitivity cardiac troponin I concentration at presentation for type 1 myocardial infarction in all patients with suspected acute coronary syndrome (blue) and in those with a primary symptom of chest pain (red). Dotted lines illustrate the positive predictive value of the uniform 99th percentile and 5-fold upper reference limit (URL).

At presentation, troponin concentrations were similar in type 1 (median [25th–75th percentile] 91 [30–493] ng/L) and type 2 (50 [22–147] ng/L) myocardial infarction and in acute (50 [26–134] ng/L) and chronic (51 [31–130] ng/L) myocardial injury (Figure 1 and Table 1). A troponin concentration above the uniform 99th percentile at presentation gave a PPV of 48% and specificity of 92% for type 1 myocardial infarction (Table 2). The sex-specific 99th percentile of 16 ng/L in women gave a PPV and specificity of 39% and 89%, whereas the sex-specific 99th percentile of 34 ng/L in men gave a PPV and specificity of 56% and 93%, respectively. The rule-in threshold of 64 ng/L and 5-fold URL threshold gave PPVs of 57% and 62%, respectively, with specificities of 96% and 97% (Figure 2). To achieve a PPV of 75%, a rule-in threshold of 1303 ng/L was required, whereas no threshold gave a PPV of ≥90%.

**Table 2.**
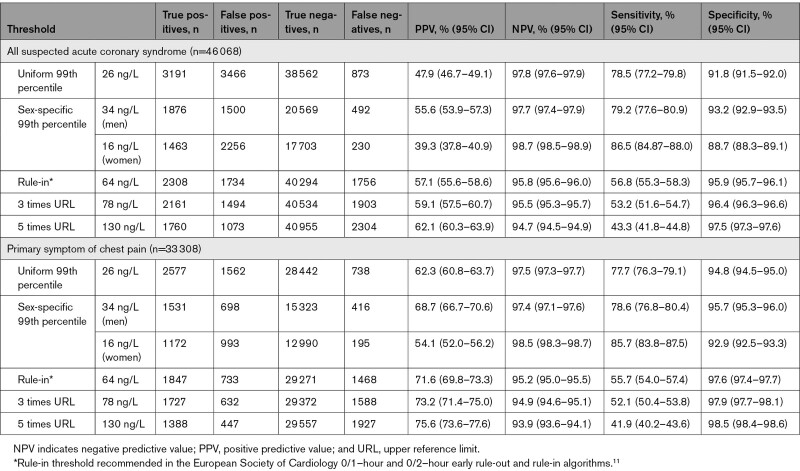
Diagnostic Performance of Cardiac Troponin Concentration at Presentation in All Patients With Suspected Acute Coronary Syndrome and in Those With a Primary Symptom of Chest Pain

In a sensitivity analysis restricted to 82% (33 319 of 40 844) of patients in whom the primary presenting symptom was chest pain (Table I in the Data Supplement), the PPV and specificity for type 1 myocardial infarction at the rule-in threshold of 64 ng/L were 72% and 98%, respectively (Table 2). The 5-fold URL threshold gave a PPV of 75% and a specificity of 99%. A rule-in threshold of 119 ng/L gave a PPV of 75%, but no threshold achieved a PPV of 90% in this population (Figure 2).

### Troponin Kinetics in Myocardial Injury and Infarction

**Figure 3.**
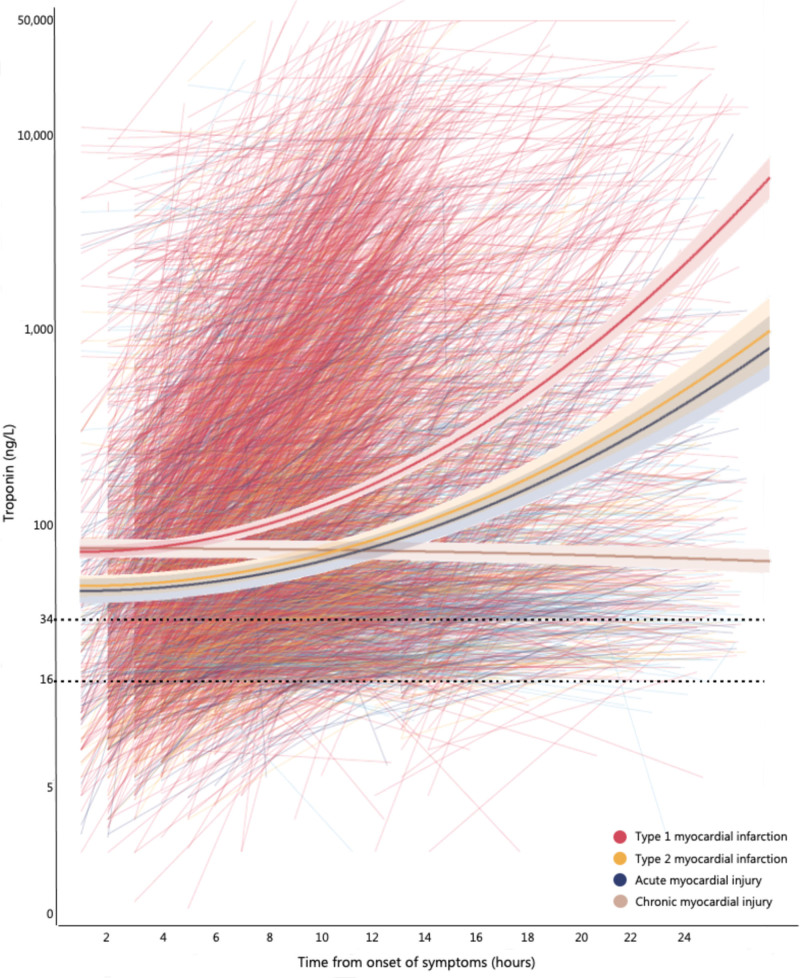
**Kinetics of high-sensitivity cardiac troponin I concentration from symptom onset in patients with myocardial injury and infarction.** Spaghetti plot illustrating high-sensitivity cardiac troponin I concentrations in relation to the time of symptom onset in individual patients stratified by the adjudicated diagnosis: type 1 myocardial infarction (red), type 2 myocardial infarction (yellow), acute myocardial injury (blue), and chronic myocardial injury (gray). Plot is restricted to those patients in whom any troponin concentration was above the sex-specific 99th percentile concentration during serial testing within 12 hours of presentation and for whom the time of symptom onset was known (n=3845). Linear mixed-effects modeling was done using random intercepts and random slopes, including quadratic terms for time, with cardiac troponin I as outcome. The output from a linear mixed-effects model incorporating time from symptom onset, troponin, and change in troponin concentration is overlaid for each condition. For each condition, the final model to estimate the trajectory of cardiac troponin I was chosen according to the Akaike information criteria.

**Figure 4.**
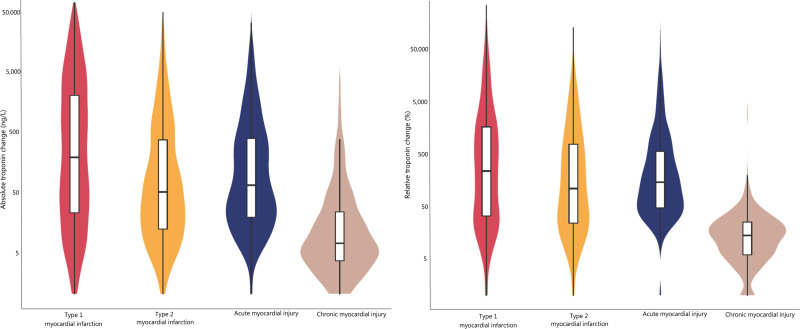
**Absolute and relative changes in high-sensitivity cardiac troponin I concentration on serial testing in patients with myocardial injury and infarction.** Violin-density and box-and-whisker plots illustrating the absolute and relative change in high-sensitivity cardiac troponin I concentration on serial testing in patients stratified by the adjudicated diagnosis: type 1 myocardial infarction (red), type 2 myocardial infarction (yellow), acute myocardial injury (blue), and chronic myocardial injury (gray).

Serial troponin testing within 12 hours of presentation was performed in 4187 patients (51%) with concentrations above the sex-specific 99th percentile. The time from symptom onset to initial troponin sampling was similar in patients with type 1 and type 2 myocardial infarction and acute myocardial injury (240 [180–420] minutes) but was longer in patients with chronic myocardial injury (300 [180–780] minutes). The rate of change in troponin within 12 hours of presentation was highest in patients with type 1 myocardial infarction compared with those with type 2 myocardial infarction and acute or chronic myocardial injury (*P*<0.001 for all; Figure 3). The absolute change in troponin concentration differed in patients with type 1 myocardial infarction (177 [21–1929] ng/L) compared with those with type 2 myocardial infarction (46 [10–365] ng/L), acute nonischemic myocardial injury (57 [17–384] ng/L), and chronic myocardial injury (6 [2–22] ng/L; *P*<0.001 for all; Figure 4). The relative change in troponin concentration also differed between patients with type 1 myocardial infarction (231% [31%–1602%]) compared with those with type 2 myocardial infarction (105% [22%–656%]), acute nonischemic myocardial injury (129% [45%–534%]), and chronic myocardial injury (12% [5%–24%]; *P*<0.001 for all).

Combining troponin concentration at presentation with an absolute change in troponin on serial testing of ≥15 ng/L or relative change of ≥20% improved discrimination for type 1 myocardial infarction compared with troponin concentration at presentation alone (0.646 [0.627–0.666] and area under the receiver operating characteristic curve, 0.661 [0.642–0.680], respectively, versus 0.613 [0.594–0.633]; Figure II in the Data Supplement).

## Discussion

In consecutive patients with suspected acute coronary syndrome, we evaluated whether troponin concentrations at presentation or their kinetics differed sufficiently to discriminate between myocardial injury and infarction. We report a number of observations that are relevant to practice. First, troponin concentrations at presentation are similar in patients with myocardial injury and those with infarction regardless of the diagnostic classification. Second, the use of recommended rule-in thresholds above the 99th percentile provides only marginal improvements in the PPV and specificity for type 1 myocardial infarction. Troponin thresholds >1000 ng/L would be required to achieve a PPV of ≥75%. Third, the magnitude and rate of change of troponin can help differentiate type 1 from type 2 myocardial infarction and acute or chronic myocardial injury. Although we observed important differences in troponin kinetics, the troponin concentration at presentation provides only limited discrimination between type 1 myocardial infarction and other causes of myocardial injury or infarction. Clinical context may be more helpful than any given rule-in threshold to guide the triage and initial management of patients with suspected acute coronary syndrome in practice.

In contrast with the previous generation of cardiac troponin assays, high-sensitivity assays are able to precisely measure troponin at very low concentrations. Accelerated diagnostic pathways that harness this enhanced precision to enable earlier decisions to rule out and rule in myocardial infarction are now used widely around the world and have been recommended by international guidelines.^1,11,32,33^ The diagnostic performance of these pathways has been validated in multiple observational studies,^16,34–38^ and the effectiveness and safety of ruling out myocardial infarction earlier have been demonstrated in randomized controlled trials.^39,40^ However, the only prior randomized trial to evaluate the effectiveness of applying the 99th percentile to rule in myocardial infarction did not demonstrate better outcomes.^41^

The major advantage of accelerated diagnostic pathways using high-sensitivity cardiac troponin testing is that they improve confidence to rule out myocardial infarction and to reduce the need for admission to hospital.^24,39,40^ However, the use of lower thresholds to diagnose myocardial infarction has identified more patients with elevated cardiac troponin concentrations attributable to other conditions.^2,3,9^ Therefore, thresholds above the 99th percentile have been proposed to improve the specificity and PPV and to accelerate the rule-in of myocardial infarction. Assay-specific rule-in thresholds are recommended by the European Society of Cardiology practice guidelines, which also advocate that patients with troponin concentrations above these thresholds at presentation be triaged to a coronary care unit and undergo coronary angiography.^11^ However, the performance of these rule-in thresholds has not been evaluated in clinical practice, where testing is often performed in a broader group of patients. Our findings are consistent with the concept that underpins these recommendations: the higher the troponin concentration at presentation, the higher the likelihood of type 1 myocardial infarction. However, the PPV of the rule-in threshold was 57%, considerably lower than the 77% and 70% reported in the derivation and validation of this rule-in threshold.^42^ Our observations are consistent with a recent study-level meta-analysis that reported that the PPV of the rule-in component of a multithreshold 0/1–hour pathway using a high-sensitivity cardiac troponin T assay was 51%.^43^ However, this was not a patient-level analysis, and the performance of the rule-in threshold in isolation was not reported. When our patient population was restricted to the 33 308 patients presenting with a primary symptoms of chest pain to enable direct comparison with those studies in which the rule-in threshold was defined, we observed a substantial improvement in the PPV to 72%. Taken together, these observations highlight the importance of interpreting cardiac troponin in context and the merits of evaluating the performance of diagnostic tests in the population in which they are applied in practice.

In consecutive patients with suspected acute coronary syndrome, half of all patients with a concentration of troponin above the sex-specific 99th percentile had a diagnosis of type 2 myocardial infarction or acute and chronic nonischemic myocardial injury. Our findings are consistent with those from the BACC study (Biomarkers in Acute Cardiac Care) in which just 29% of patients with an elevated cardiac troponin T concentration had a diagnosis of type 1 myocardial infarction.^44^ Although we observed that troponin concentrations were higher in type 1 myocardial infarction, there was substantial overlap with type 2 myocardial infarction and myocardial injury, suggesting that troponin alone at any threshold cannot reliably discriminate between these conditions. Even at a threshold 5 times the URL, purported to have a PPV of >90%,^11^ we observed that the PPV was just 62% for type 1 myocardial infarction. A threshold >50 times the URL would be required to achieve a PPV of 75% when applied to all patients with suspected acute coronary syndrome.

Although our observations highlight the limitations of using single troponin measurements to triage patients, confirmation of the diagnosis of myocardial infarction requires serial testing and a rise or fall in cardiac troponin.^1^ We observed differences in the rate of troponin release with a higher rate of change, as well as larger absolute and relative changes on serial sampling, in patients with type 1 myocardial infarction compared with those with type 2 myocardial infarction or acute and chronic myocardial injury. Despite these differences, the use of relative or absolute delta change criteria only marginally improved discrimination compared with the troponin concentration alone. This is perhaps not surprising given the observed changes in troponin concentration on serial testing in both type 2 myocardial infarction and acute myocardial injury. Although previous studies using a contemporary sensitive troponin assay in a small cohort of 66 and 188 patients with type 1 and type 2 myocardial infarction, respectively, suggested no improvement in discrimination when change in troponin at 3 or 6 hours was combined with the absolute concentration,^45^ we observed a modest improvement. It would seem unlikely that the shorter time intervals between serial testing would improve discrimination, but this should be evaluated in future studies.

In recent years, a number of approaches have been proposed that could enable clinicians to use cardiac troponin more flexibly.^31,46,47^ These approaches recognize the limitations of applying fixed thresholds to triage a heterogeneous population of patients and the challenge of performing serial testing at precise intervals in clinical practice. The Troponin Only–Manchester Acute Coronary Syndrome rule uses logistic regression to provide individual patient risk estimates for non–ST-segment–elevation myocardial infarction by incorporating age, sex, clinical variables, and a measure of high-sensitivity cardiac troponin T at presentation. This rule performs well but does not discriminate type 1 myocardial infarction from other causes of troponin elevation or take into account serial testing.^47^ In a collaborative analysis that pooled data from multiple cohorts, the COMPASS-MI (Calculation of Myocardial Infarction Risk Probabilities to Manage Patients With Suspicion of Myocardial Infarction) investigators highlight that a more flexible approach is required and demonstrate proof of concept that the negative predictive value and PPV for type 1 myocardial infarction vary across a range of thresholds and delta change in troponin values.^46^ Finally, the myocardial ischemic injury index uses a gradient-boosting machine learning algorithm to combine age, sex, and paired high-sensitivity cardiac troponin I values to compute a value (0–100) that reflects the likelihood of type 1 myocardial infarction for an individual patient.^31^ Serial testing can be performed at any time point, and the algorithm incorporates a measure of rate of change in troponin. Although each of these approaches shows considerable promise, it is unclear at present whether the use of these probabilistic scores in practice improves clinical decisions compared with existing guideline-recommended pathways using fixed thresholds.

We recognize some strengths and limitations of this study. First, we enrolled consecutive patients in whom the attending clinician suspected acute coronary syndrome by embedding our screening tool into the electronic health care system. This avoided selection bias and ensured that our study population was representative. Second, all diagnoses were adjudicated according to the Fourth Universal Definition of Myocardial Infarction, ensuring that our findings are relevant to contemporary practice. Although few patients had missing troponin values for the evaluation of rule-in thresholds (0.1%), serial testing was performed at the discretion of the attending clinician and was performed in only 53% of patients with myocardial injury or infarction. It is likely that those patients undergoing serial testing differed from those who had a single test performed; however, our comparison between patients with type 1 and type 2 myocardial infarction and those with myocardial injury was limited to the subgroup of patients with ≥2 tests performed within 12 hours of presentation. Cardiac troponin was measured with a single high-sensitivity cardiac troponin I assay, and we recognize that the performance of rule-in thresholds for myocardial infarction is likely to differ for other assays. Last, the approach to patient selection for cardiac troponin testing will vary across health care systems, and we recommend some caution in extrapolating the performance of rule-in thresholds to sites where testing is performed more widely.

## Conclusions

Although we observed important differences in the kinetics, cardiac troponin concentrations at presentation are insufficient to distinguish type 1 myocardial infarction from other causes of myocardial injury or infarction in practice. Clinical context may be more helpful than any rule-in threshold for guiding initial triage and management decisions.

## Acknowledgments

R.W., F.S.A., P.O.C., D.M.K., A.R.C., and N.L.M. conceived the study and its design. R.W., D.M.K., and A.R.C. had access to the data and performed the analysis. R.W., D.M.K., A.R.C., and N.L.M. interpreted the data and drafted the article. All authors revised the article critically for important intellectual content and provided their final approval of the version to be published. All authors are accountable for the work. The authors thank the High-STEACS Investigators for their contributions to the conception or design of the work, or the acquisition, analysis, or interpretation of data for the work.

## Sources of Funding

The High-STEACS trial was funded by a Special Project Grant from the British Heart Foundation (SP/12/10/29922). Dr Wereski, Dr Bularga, and D. Doudesis are supported by Clinical Research Training Fellowships (MR/V007017/1, MR/V007254/1) and a PhD studentship (MR/N013166/1) from the Medical Research Council. Dr Lee is supported by a Clinical Research Training Fellowship (FS/18/25/33454) from the British Heart Foundation. Dr Kimenai is supported by a grant from Health Data Research UK, which receives its funding from Health Data Research UK Ltd (HDR-5012) funded by the UK Medical Research Council, Engineering and Physical Sciences Research Council, Economic and Social Research Council, Department of Health and Social Care (England), Chief Scientist Office of the Scottish Government Health and Social Care Directorates, Health and Social Care Research and Development Division (Welsh Government), Public Health Agency (Northern Ireland), British Heart Foundation, and Wellcome Trust. Dr Chapman receives support from a Starter Grant for Clinical Lecturers by the Academy of Medical Sciences (SGL021/1075). Dr Mills is supported by the Butler British Heart Foundation Senior Clinical Research Fellowship (FS/16/14/32023) and a Program Grant (RG/20/10/34966) and Research Excellence Award (RE/18/5/34216) from the British Heart Foundation. Abbott Laboratories provided cardiac troponin assay reagents, calibrators, and controls without charge. The funders played no role in the design and conduct of the study; collection, management, analysis, and interpretation of the data; preparation, review, or approval of the manuscript; and decision to submit the manuscript for publication.

## Disclosures

Dr Apple reports research grants awarded to the Minneapolis Medical Research Foundation from Abbott Diagnostics, Siemens Diagnostics, Ortho-Clinical Diagnostics, and Beckman Coulter outside the submitted work and personal fees from HyTest Ltd. Dr Mills has received honoraria from Abbott Diagnostics, Siemens Healthineers, Roche Diagnostics, and LumiraDx, and the University of Edinburgh has received research grants from Abbott Diagnostics and Siemens Healthineers. The other authors report no conflicts.

## Supplemental Materials

Expanded Methods

Supplemental Figures I and II

Supplemental Table I

## Supplementary Material


