# Supplementary material for: Cardiac Troponin Thresholds and Kinetics to Differentiate Myocardial Injury and Myocardial Infarction
Source: Circulation. 2021 Jun 25;144(7):528–38. doi: 10.1161/CIRCULATIONAHA.121.054302 (PMC8360674; doi:10.1161/CIRCULATIONAHA.121.054302)
Supplement: Supplementary file 1 [file cir-144-528-s001.pdf]

SUPPLEMENTARY MATERIAL

Cardiac troponin thresholds and kinetics to differentiate myocardial injury and myocardial infarction

Ryan Wereski MD[1*], Dorien M Kimenai PhD[2*], Caelan Taggart MD[1], Dimitrios Doudesis MSc[1,2], Kuan Ken Lee MD[1], Matthew TH Lowry MD[1], Anda Bularga MD,[1] David J Lowe MD[3], Takeshi Fujisawa PhD[1], Fred S. Apple PhD[4], Paul O Collinson MD PhD[5], Atul Anand MD PhD[1], Andrew R Chapman MD PhD[1], Nicholas L Mills MD PhD[1,2]

[1] British Heart Foundation Centre for Cardiovascular Science, University of Edinburgh, Edinburgh, UK
[2] Usher Institute, University of Edinburgh, Edinburgh, UK
[3] University of Glasgow, School of Medicine, Glasgow, Scotland
[4] Department of Laboratory Medicine and Pathology, Hennepin Healthcare/Hennepin County Medical Center and University of Minnesota, Minneapolis, USA
[5] Department of Clinical Blood Sciences and Cardiology, St George's University of London, London, UK

* Contributed equally

Corresponding author:
Professor Nicholas L. Mills
BHF/University Centre for Cardiovascular Science
Royal Infirmary of Edinburgh
University of Edinburgh
Edinburgh EH16 4SA
United Kingdom

Telephone: +44 131 242 6515
Email: nick.mills@ed.ac.uk

Running title: Troponin release kinetics

Twitter: @HighSTEACS, @RyanWereski, @chapdoc1

Additional Methods

Detailed description of diagnostic adjudication

All patients with hs-cTnI concentrations above the sex-specific 99th percentile were classified according to the Third Universal Definition of Myocardial Infarction in use at the time of the trial. In this pre-specified secondary analysis, we updated this classification in accordance with the Fourth Universal Definition of Myocardial Infarction. The final diagnosis was adjudicated according to a pre-specified list (cardiac diagnoses: acute aortic dissection, acute heart failure, cardiomyopathy, chronic heart failure, hypertensive heart disease, myopericarditis, non-ST segment elevation myocardial infarction, ST-segment elevation myocardial infarction, recent myocardial infarction, tachyarrhythmia, Takotsubo cardiomyopathy or valvular heart disease; non-cardiac diagnoses: acute kidney injury, chronic kidney disease, chronic obstructive pulmonary disease, gastrointestinal bleed, pulmonary embolism, sepsis, or other). Two physicians independently reviewed all clinical information, blinded to study phase, with discordant diagnoses resolved by a third reviewer. Clinical information included the dates and times of presentation and final discharge, the initial emergency department assessment and final discharge letter as documented in the electronic care record, with summaries of all investigations undertaken during the index presentation including the electrocardiogram. The adjudication panel had access to raw clinical information including haemoglobin, creatinine and high-sensitivity cardiac troponin I concentrations, and the reports from invasive coronary angiography. Type 1 myocardial infarction was defined as myocardial necrosis (any hs-cTnI concentration above the 99th percentile with a rise and/or fall in hs-cTnI concentration where serial testing was performed) in the context of a presentation with suspected acute coronary syndrome with symptoms or signs of myocardial ischemia on the electrocardiogram. Patients with symptoms or signs of myocardial ischemia and evidence of increased oxygen demand or

decreased supply (for example, tachyarrhythmia, hypotension, or anaemia) secondary to an alternative pathology and myocardial necrosis were defined as type 2 myocardial infarction. The classification of type 2 myocardial infarction also includes patients with coronary vasospasm, embolism or spontaneous dissection without evidence of atherothrombosis related to coronary artery disease. Type 4a myocardial infarction was defined in patients with symptoms or signs of myocardial ischemia following percutaneous coronary intervention where hs-cTnI concentrations were 5-fold greater than the 99th percentile, or increased further if elevated prior to the procedure. Type 4b myocardial infarction was defined where myocardial ischemia and myocardial necrosis were associated with stent thrombosis documented at angiography. Myocardial injury was defined if hs-cTnI concentrations were above the 99th percentile in the absence of any clinical features of myocardial ischemia. All non-ischemic myocardial injury was classified as acute, unless a change of <20% was observed on serial testing or the final adjudicated diagnosis was chronic heart failure or chronic renal failure, where the classification was chronic myocardial injury.

Supplemental Table

Table I. Baseline characteristics of patients stratified by primary presenting complain of chest pain or other symptoms

	Overall	Primary presentation with chest pain	Other primary symptom
**No. of participants**	46,092	33,319	7,525
Age(years)	61.0 (49.0 - 75.0)	58.0 (47.0 - 72.0)	71.0 (58.0 - 81.0)
Sex (Male)	24,433 (53.0)	17,967 (53.9)	3,837 (51.0)
Adjudicated diagnosis			
Type 1 myocardial infarction	33,319 (81.6)	3,315 (10.0)	377 (5.0)
Type 2 myocardial infarction	1,977 (4.8)	744 (2.2)	282 (4.7)
Acute myocardial injury	2,003 (4.9)	569 (1.7)	926 (12.3)
Chronic myocardial injury	1,213 (3.0)	559 (1.7)	572 (7.6)
No myocardial injury	2,332 (5.7)	28,080 (84.3)	5,361 (71.3)
Past medical history			
Coronary artery disease	11,349 (24.6)	8,046 (24.2)	1,824 (24.2)
Myocardial infarction	4,003 (8.7)	2,818 (8.5)	623 (8.3)
Diabetes mellitus	3,274 (7.1)	2,283 (6.9)	587 (7.8)
Cerebrovascular disease	2,732 (5.9)	1,709 (5.1)	667 (8.9)
Hypercholesterolaemia	18,412 (39.9)	12,641 (38.0)	3,521 (46.8)
Heart failure	3,908 (8.5)	2,377 (7.1)	1,035 (13.8)
Abnormal renal function	8,398 (18.7)	4,869 (15.0)	2,456 (33.4)
Previous revascularisation			
PCI	3,543 (7.7)	2,686 (8.1)	456 (6.1)
CABG	747 (1.6)	511 (1.5)	149 (2.0)
Electrocardiogram*			
Normal	2,522 (37.3)	1,844 (40.6)	458 (27.4)
Myocardial ischemia	1,740 (25.7)	1,277 (28.1)	369 (22.1)
ST depression	1,185 (17.5)	858 (18.9)	248 (14.8)
ST elevation	243 (3.6)	179 (3.9)	56 (3.4)

T-wave inversion	1,191 (17.6)	831 (18.3)	272 (16.3)
Observations, haematology, and clinical chemistry			
Systolic blood pressure, mmHg	139.4 (28.9)	140.5 (27.8)	136 (31.9)
Heart rate, bpm	85.9 (26.4)	82.7 (24.2)	94 (30.4)
Haemoglobin, g/L	137.2 (18.2)	138.0 (17.5)	134.6 (19.9)

Presented as No. (%), mean±SD or median [25th percentile – 75th percentile]. Abbreviations: CABG = coronary artery bypass grafting; PCI = percutaneous coronary intervention. *Electrocardiographic data reported for the 15% (6,218/40,829) of patients with myocardial infarction or myocardial injury who had electrocardiographic and symptom onset data available.

Figure I.

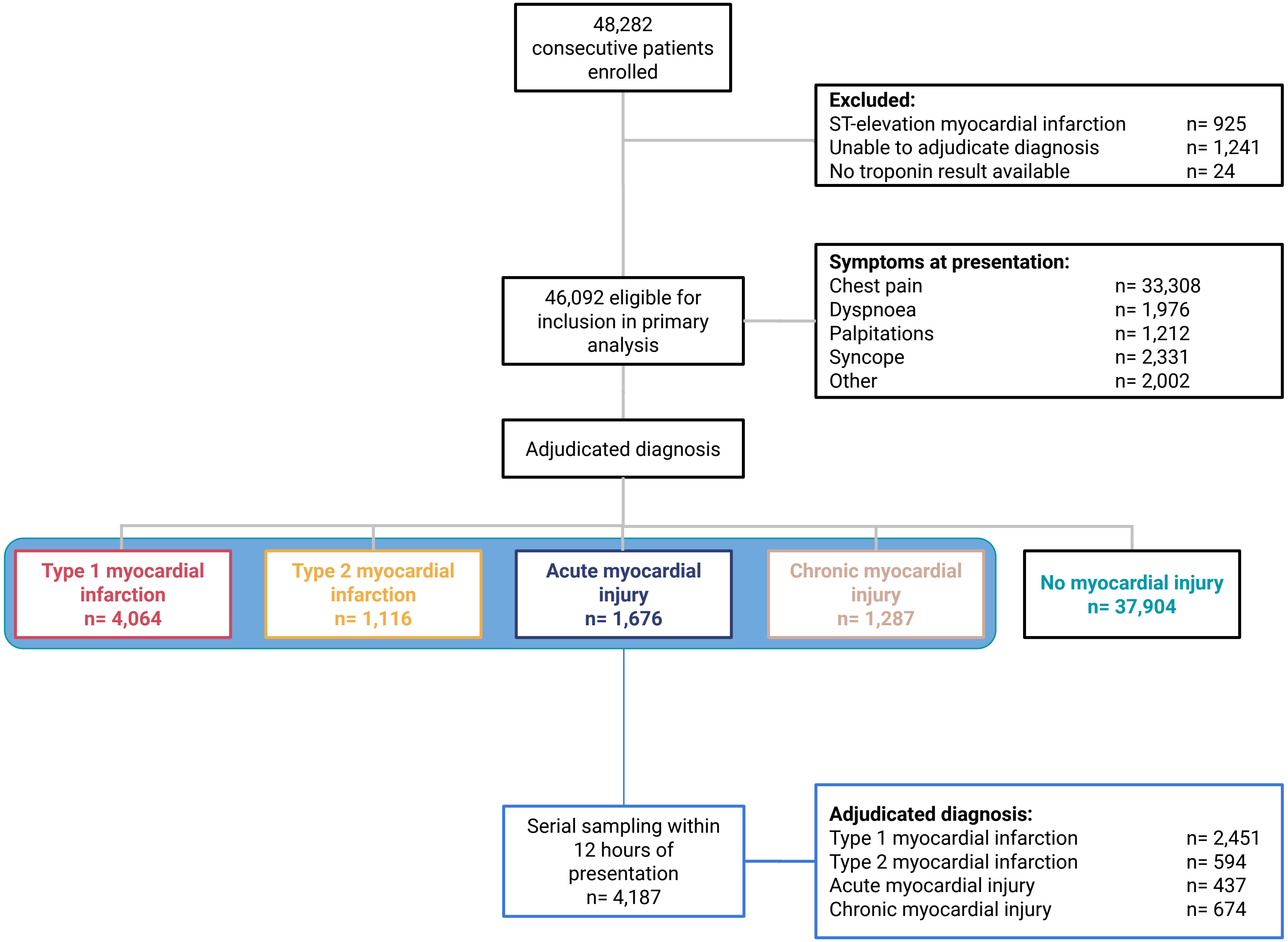
48,282 consecutive patients enrolled
Excluded:
ST-elevation myocardial infarction n= 925
Unable to adjudicate diagnosis n= 1,241
No troponin result available n= 24
46,092 eligible for inclusion in primary analysis
Symptoms at presentation:
Chest pain n= 33,308
Dyspnoea n= 1,976
Palpitations n= 1,212
Syncope n= 2,331
Other n= 2,002
Adjudicated diagnosis
Type 1 myocardial infarction n= 4,064
Type 2 myocardial infarction n= 1,116
Acute myocardial injury n= 1,676
Chronic myocardial injury n= 1,287
No myocardial injury n= 37,904
Serial sampling within 12 hours of presentation n= 4,187
Adjudicated diagnosis:
Type 1 myocardial infarction n= 2,451
Type 2 myocardial infarction n= 594
Acute myocardial injury n= 437
Chronic myocardial injury n= 674

Figure II.

A

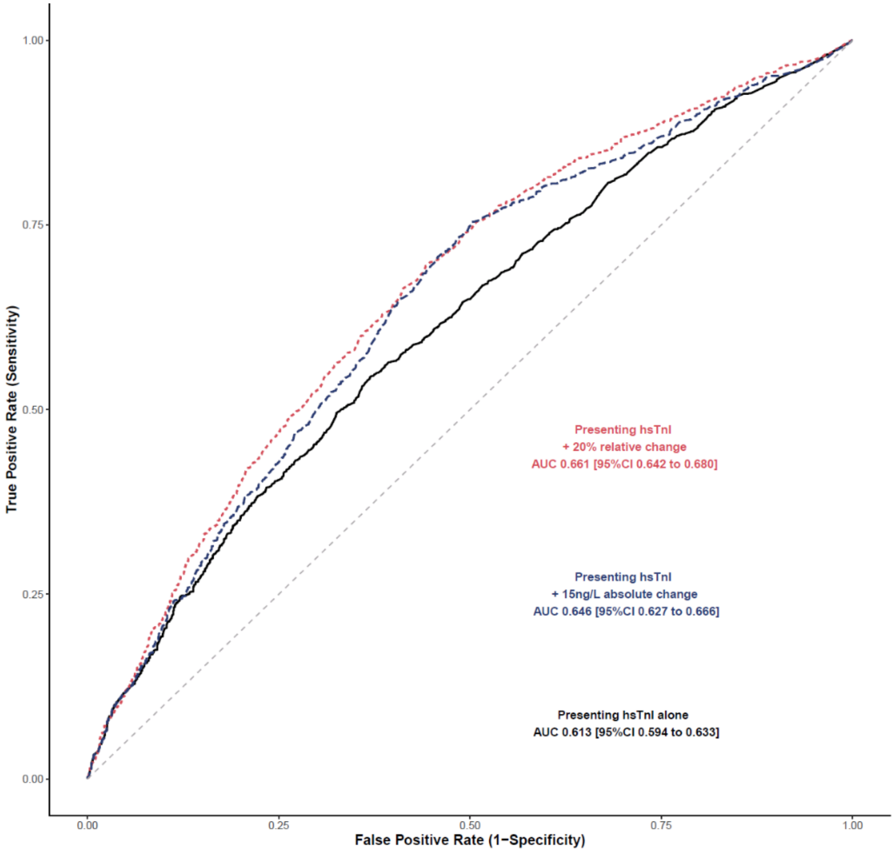
True Positive Rate (Sensitivity)
1.00
0.75
0.50
0.25
0.00
Presenting hsTnI
+ 20% relative change
AUC 0.661 [95%CI 0.642 to 0.680]
Presenting hsTnI
+ 15ng/L absolute change
AUC 0.646 [95%CI 0.627 to 0.666]
Presenting hsTnI alone
AUC 0.613 [95%CI 0.594 to 0.633]
0.00
0.25
0.50
0.75
1.00
False Positive Rate (1−Specificity)

B

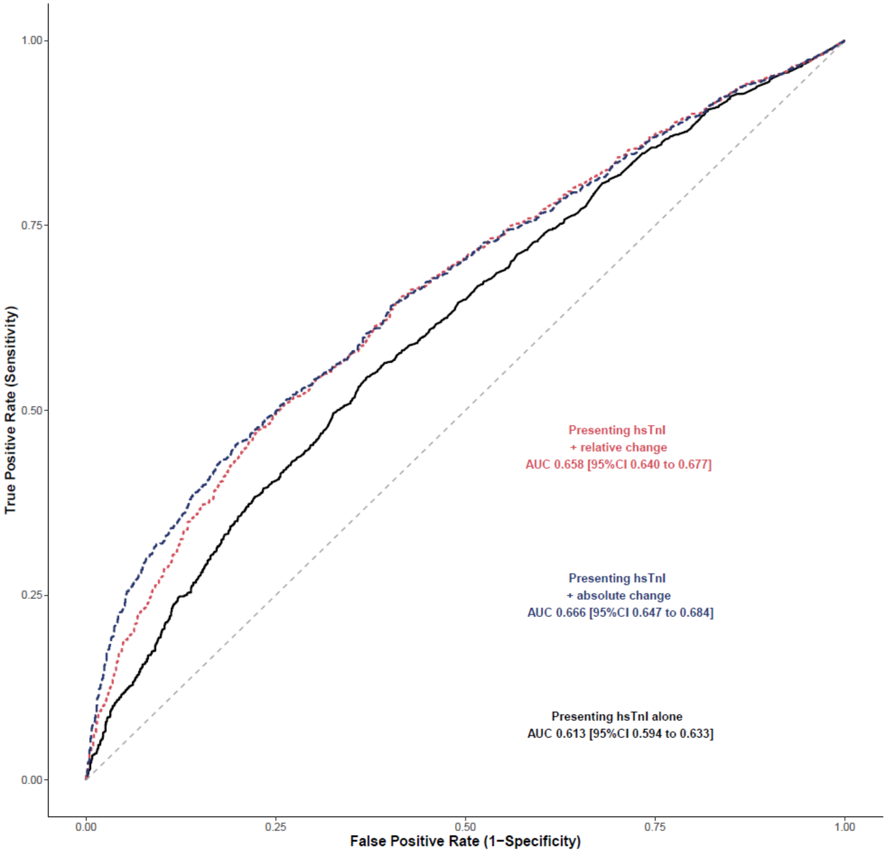
True Positive Rate (Sensitivity)
1.00
0.75
0.50
0.25
0.00
Presenting hsTnI
+ relative change
AUC 0.658 [95%CI 0.640 to 0.677]
Presenting hsTnI
+ absolute change
AUC 0.666 [95%CI 0.647 to 0.684]
Presenting hsTnI alone
AUC 0.613 [95%CI 0.594 to 0.633]
0.00
0.25
0.50
0.75
1.00
False Positive Rate (1−Specificity)

Supplemental Figures

Figure I. Flow-Chart of the Study and Analysis Population

Figure II. Discrimination of High-Sensitivity Cardiac Troponin I at Presentation for Type 1 Myocardial Infarction With and Without an Absolute and Relative Change in Cardiac Troponin Concentration

Comparison of the discrimination of high-sensitivity cardiac troponin I concentration at presentation in isolation (black) and in combination with a relative change in concentration (red), or in combination with an absolute change in concentration (blue) to distinguish type 1 myocardial infarction from other causes of myocardial injury or infarction in patients with a troponin value above the sex-specific 99th percentile upper reference limit at presentation. Panel A compares relative and absolute thresholds of 15 ng/L and 20% respectively, and Panel B compares absolute and relative change on a continuous scale.
